# The Use of EST Expression Matrixes for the Quality Control of Gene Expression Data

**DOI:** 10.1371/journal.pone.0032966

**Published:** 2012-03-08

**Authors:** Andrew T. Milnthorpe, Mikhail Soloviev

**Affiliations:** School of Biological Sciences, CBMS, Royal Holloway University of London, Egham, Surrey, United Kingdom; University of North Carolina at Charlotte, United States of America

## Abstract

EST expression profiling provides an attractive tool for studying differential gene expression, but cDNA libraries' origins and EST data quality are not always known or reported. Libraries may originate from pooled or mixed tissues; EST clustering, EST counts, library annotations and analysis algorithms may contain errors. Traditional data analysis methods, including research into tissue-specific gene expression, assume EST counts to be correct and libraries to be correctly annotated, which is not always the case. Therefore, a method capable of assessing the quality of expression data based on that data alone would be invaluable for assessing the quality of EST data and determining their suitability for mRNA expression analysis. Here we report an approach to the selection of a small generic subset of 244 UniGene clusters suitable for identification of the tissue of origin for EST libraries and quality control of the expression data using EST expression information alone. We created a small expression matrix of UniGene IDs using two rounds of selection followed by two rounds of optimisation. Our selection procedures differ from traditional approaches to finding “tissue-specific” genes and our matrix yields consistency high positive correlation values for libraries with confirmed tissues of origin and can be applied for tissue typing and quality control of libraries as small as just a few hundred total ESTs. Furthermore, we can pick up tissue correlations between related tissues e.g. brain and peripheral nervous tissue, heart and muscle tissues and identify tissue origins for a few libraries of uncharacterised tissue identity. It was possible to confirm tissue identity for some libraries which have been derived from cancer tissues or have been normalised. Tissue matching is affected strongly by cancer progression or library normalisation and our approach may potentially be applied for elucidating the stage of normalisation in normalised libraries or for cancer staging.

## Introduction

EST expression profiling has by now become well-established high-throughput method for acquiring quantitative information on a sample's transcriptome and for studying differential gene expression, inferred from the differences in the relative numbers of EST tags between two libraries. To facilitate gene discovery, the EST content of a library can be altered to reduce the abundance of transcripts representing genes with high expression. To achieve this a library can be either normalised by removing the most abundant transcripts in order to reduce or eliminate the differences in the relative transcript abundances to a narrow range [1]–[4], or subtracted to enrich the library for rare novel transcripts [5], [6]. Ideally this should create a library containing the same or similar tag counts for the low abundance sequences as before, but with vastly reduced counts for abundant or unwanted cDNAs. Neither normalised nor subtracted libraries are suitable for studying differential mRNA expression because of the significantly changed representation or removal of the original transcripts.

Growing amounts of EST as well as SAGE and microarray data triggered off growth of large databases for storage, processing and retrieval of the data. For example the current version of the Cancer Genome Anatomy Project (CGAP) database contains 2,507,631 individual records and 6,694,344 EST counts for Homo sapiens covering 122,755 individual UniGene clusters. (http://cgap.nci.nih.gov/Info/CGAPDownload, accessed 15th July 2011). The CGAP database contains EST tag counts from *Homo sapiens* or *Mus musculus* EST libraries and provides the information and data mining tools needed to elucidate the molecular anatomy of cancerous cells from the UniGene repository [7], [8]. The database has been used in investigations into differential gene expression into a wide variety of cancers, for example, breast cancer [9], colon cancer [10], [11], gastric cancer [12], lung cancer [13], prostate cancer [14].

EST data, library annotations and analysis algorithms may contain errors. These include clustering errors [15], [16], annotation errors and data retrieval errors [17]. The methods used to generate cDNA libraries such as RT-PCR and fractionation of cDNAs can also introduce biases into EST data [18], [19]. However, because of a variety of potential experimental and annotation errors and the quality of EST data, normalisation and subtraction issues, one cannot be sure whether libraries used are suitable for quantitative mRNA expression analysis or not. Library tissue origins are not always known or correctly reported and some libraries originate from pooled or mixed tissue samples. Therefore, a method capable of assessing the quality of expression data based on the library expression data alone would be invaluable for assessing the quality of EST expression data and determining their suitability for mRNA expression analysis. The aim of this investigation was to produce an objective and easy to follow methodology for identifying the tissue of origin for EST libraries and for evaluating the quality of EST expression data and their suitability for digital gene expression analysis.

We hypothesised that the expression levels of a set of tissue-specific markers could be used to determine the quality of EST expression data, to verify the identity of known libraries or to identify unknown libraries. In the current investigation we used human EST expression data to find a set of markers for determining tissue specificity of EST libraries. We chose to use libraries from the CGAP database (http://cgap.nci.nih.gov/) because of the wide use of this repository for studying differential gene expression in cancer.

## Methods

### Selection of tissue specific UniGene clusters

Candidate tissue-specific UniGene clusters were selected based on a number of criteria. Firstly, the CGAP database was manually searched for the highly abundant and tissue-specific UniGene clusters defined by their EST counts, for all individual tissue types available using the DGED tool (http://cgap.nci.nih.gov/Tissues/GXS). Separate searches were conducted for “Normal” and “Cancer” histology for all tissue types. The minimum number of sequences per library was set to 10, the tissue preparation was set to “bulk” and the library protocol to “non-normalised” in all searches. The EST library group was set to “All”, which included all CGAP, MGC, ORESTES and un-annotated libraries, the latter constituted the vast majority (∼72%) of all the libraries used. The gene lists were downloaded from CGAP and then searched for the UniGene clusters with the high odds ratio (i.e. the normalised EST abundance in the selected tissue type divided by normalised abundance in all other libraries, typically above 10), which was also statistically significant (typically P<0.05). Additional selection criteria were high relative EST expression levels in the targeted tissue (typically above 0.1% of all the ESTs counts) and low expression levels in the rest of tissue types (typically below 0.1% of all the ESTs counts). Where possible only ESTs identified in at least two libraries and counted at least three times in the tissue studied were selected. Up to thirty individual UniGene cluster IDs having the highest odds ratios and meeting all of the above criteria were selected from each of the individual tissue types. Where less than thirty or none were available, the selection criteria were relaxed and the UniGene clusters which satisfied most of the search criteria were selected (Supplementary Dataset S1). All the UniGene cluster IDs were combined (totalling 2,295 from all tissues types) and the duplicates were removed, yielding 1,089 individual UniGene cluster IDs (Supplementary Dataset S2).

The second round of search for additional tissue markers was on the basis of their absolute abundance level only. For this EST counts for each of the 37,575 different UniGene clusters from 155 non-normalised libraries from all non-cancerous tissue types were determined. Expression thresholds were set at 1,2,4,8,10,12,14,16,18,20,22,24,26,28,30,32,63,128 and 256, and subsets of genes based on their maximum expression level recorded across all these libraries (across all the tissues) were identified. Statistical relationships between these subsets and the previously constructed list of 1,089 genes were identified. The maximum positive correlation value of +0.48 was recorded for the subset of genes with the maximum EST counts of at least 18 in at least one of the 155 libraries tested (Figure S1). That subset contained 909 UniGene IDs, of which 483 were already among the earlier found genes (the 1,089 set). The newly identified 426 UniGene IDs were added to the original selection yielding 1,515 UniGene IDs (Dataset S3). Following a more recent update this list was reduced to 1,437 UniGene IDs by excluding 78 cluster IDs (due to removal of these entries from the subsequent CGAP database release) (Dataset S3). We then calculated expression levels (EST counts) for each of these 1,437 UniGene clusters for each of the main 26 human tissues matching tissue definitions of CGAP database, except for bone marrow, which was combined with bone, its parent tissue, and cerebellum and cerebrum, which were combined with brain, of which they are dependent tissues. However, some tissues, e.g. brain and nearby pituitary gland were not combined because despite being close together, therefore relevant EST libraries were assigned to different tissues. Also, having a few tissues with only limited (often single) suitable EST libraries would not allow the consistent analysis of all dependent tissues at many levels of resolution. To avoid such inconsistency, we did not analyse dependent tissues. The produced expression matrix (1,437 UniGene cluster IDs×26 tissues) was used for further optimisation.

### Optimised selection of UniGene clusters

The first round of optimisation aimed to reduce inter-tissue correlations. Tissue-specific expression “super-libraries” were created for 26 tissues from 126 bulk, non-normalised libraries made from normal tissues with at least 200 total EST counts, by combining EST counts for the selected set of 1,437 UniGene cluster IDs from the same tissue, where more than one EST library per tissue was available. Pearson product-moment correlation coefficients were calculated for all pair-wise combinations of such tissue specific expression data sets. The Pearson correlation is invariant to the changes in location and scale in the variables, the calculated correlation coefficients yield comparable values within the same scale interval (−1 to +1) for all tissues and libraries irrespective of their size, coverage, the number of ESTs or any preceding linear data transformations. Sum of squared errors was used as a measure of discrepancy between the calculated correlation data and the model (no inter-tissue correlation of expression data for the selected markers). We then tested how the inter-tissue correlation values changed following removal of individual cluster expression data from the subset of 1,437 clusters. The individual UniGene clusters, removal of which had a favourable effect on the reduction of the overall inter-tissue correlations, were permanently removed and the iterative rounds of Cluster removal were repeated. The best remaining UniGene IDs (the last 505 clusters) were used for the second optimisation round, which was aimed to improve intra-tissue correlations. EST counts for each of the remaining 505 UniGene clusters for each individual non-normalised library from normal (non-cancer) tissues (the same libraries as used before) were compared to each other. This time we used individual library expression data (not the super-libraries) to calculate sum of squared differences between the calculated correlation data and the model (high intra-tissue correlation of expression data for the tissues where two or more individual libraries were available). We then tested how such intra-tissue correlation values changed following removal of individual cluster expression data from the subset of 505 clusters. After repeating this procedure for all of the 505 remaining clusters, all the clusters were scored and the ones, removal of which improved the correlations most were permanently removed. 244 UniGene IDs were eventually selected as the generic EST expression tissue-specific dataset (Dataset S3). The reduced expression matrix (244 UniGene cluster IDs×26 tissues, referred to as EST expression matrix (Dataset S4)) was used for all subsequent analyses.

## Results

### Tissue specific UniGene clusters and EST expression matrix

We hypothesised that to be suitable for the role of universal tissue specific markers, the transcripts should be (i) highly abundant in their target tissues relative to all the other tissues, (ii) should be abundant in absolute terms in target tissues. The high relative abundance (high odds ratio) defines the tissue specificity. The high absolute abundance (above 0.1%) was chosen to ensure that such transcripts would still be found even in smaller libraries with small number of total EST counts. Up to thirty individual UniGene clusters were eventually selected using criteria described in Methods section, from each of the individual tissue types. Of the 1,089 genes identified (Dataset S2), 1,044 were present in normal (non-cancer) tissues (although non-exclusively) and 479 originated from more than one tissue type. Whilst we allowed that, a further optimisation of the selected subset was necessary.

For the majority of the tissues, the original selection was made based on the very small number of libraries available in CGAP for those tissues (typically 2–4 libraries, with brain and placenta being exceptions where more than 10 libraries were available (Dataset S1)). Because of that and also because of the stringent selection requirements, it was reasonable to assume that some suitable genes could have been omitted because of the very limited choice of libraries available for the analysis and not because of them being unsuitable tissue markers. We therefore searched for additional candidate genes by looking solely into individual EST counts for all of the 37,575 different UniGene clusters from 155 non-normalised libraries from all non-cancerous tissue types. Following the procedures outlined in Methods we expanded the list of potential tissue markers to include 1,437 UniGene clusters (Dataset S3).

Because of the relaxed criteria used for selecting the potential tissue markers, in order to find the best makers and to reduce the list to a more manageable and shorter list we attempted to optimise the selection using new selection criteria independent of the ones used in the original rounds of selection. The first round of optimisation aimed to reduce inter-tissue correlations and yielded a reduced set of 505 UniGene clusters. The final optimisation round aimed to improve intra-tissue correlations. The optimised set of tissue-specific markers contained 244 UniGene cluster IDs (Dataset S3) for which EST expression matrix (244 UniGene IDs×26 tissues) was created (Dataset S4).

### Inter-tissue correlations and intra-tissue correlations using EST expression matrix

**Figure 1:**
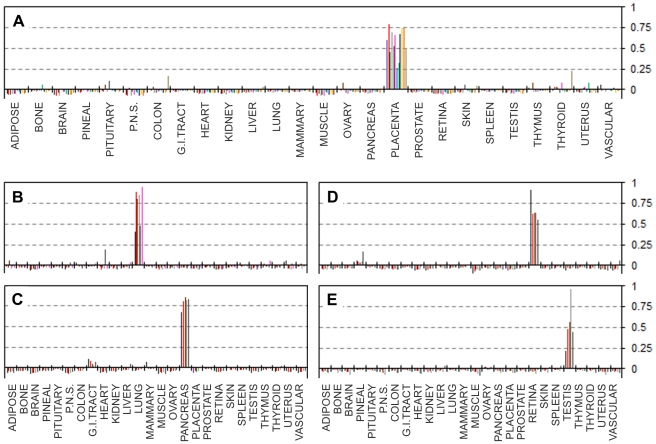
Correlation of the EST matrix with individual libraries from matching tissues showing no inter-tissue correlation. Pearson product-moment correlation coefficients (vertical axes) calculated for each of the individual EST libraries and the EST expression matrix (Supplementary Dataset S4). **A:** Placental libraries. **B:** Lung libraries. **C:** Pancreatic libraries. **D:** Retinal libraries. **E:** Testis libraries. See Supplementary Dataset S5. for the libraries' IDs.

**Figure 2:**
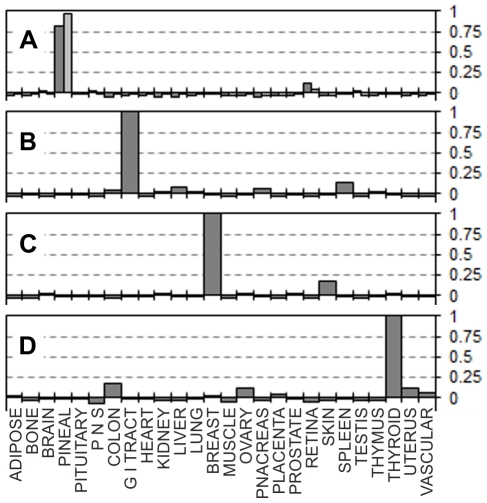
Correlation of the EST expression matrix with tissues with one or two libraries were available. Pearson product-moment correlation coefficients (vertical axes) calculated for each of the individual EST libraries and the EST expression matrix (Supplementary Dataset S4). **A:** “Soares_pineal_gland_N3HPG” library (dark bars), “Pineal gland II” (lighter bars). **B:** “Small intestine I” EST library. **C:** “NCI_CGAP_Br7” library from mammary gland. **D:** “Thyroid” EST library.

**Figure 3:**
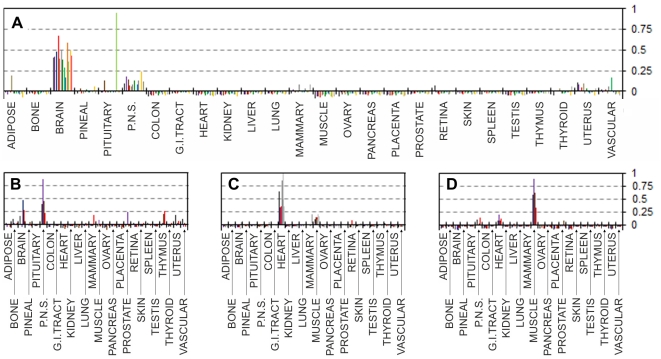
Correlation of the EST expression matrix with individual EST libraries from related tissues. Pearson product-moment correlation coefficients (vertical axes) calculated for each of the individual EST libraries and the EST expression matrix (Supplementary Dataset S4). **A:** Brain EST libraries, these include one cerebellum and one cerebrum EST libraries. Assumed mixed tissue brain library showing positive correlation with pituitary gland is “NIH_MGC_181”. **B:** Peripheral nervous system libraries showing a degree of positive correlation with brain libraries. **C:** Heart libraries showing a degree of positive correlation with muscle libraries. **D:** Muscle libraries showing a degree of positive correlation with heart libraries. See Supplementary Dataset S5. for the libraries' IDs.

Correlation values between tissue expression profiles of the 244 UniGene Clusters from the EST expression matrix and the relevant EST counts from 113 largest libraries (Dataset S5) representing 26 main human tissues were calculated. The correlation data fell into three main categories. The first group contained groups of libraries for which virtually no inter-tissue correlation was found, and where all the libraries shown good positive correlation (values ranging approximately within +0.2 to +1) with the relevant source tissues but not with any of the other tissues. Figure 1 summarises the results for five such representative tissues where correlation levels clearly confirm the identity of each of the individual EST libraries. The second group contained tissues for which only one or two non-normalised bulk EST libraries were available. In the former case (one library per tissue) positive correlations of +1 were expected, because for these tissues only our EST matrix was based on those expression data. Nevertheless, no other tissues having positive correlation above ∼0.2 were identified, confirming the absence of cross-tissue correlations for the EST matrix entries (Figure 2). The third group included tissues with some degree of multiple tissue positive correlations. These were brain tissue libraries which shown partial positive correlation with peripheral nervous system EST libraries, the peripheral nervous system libraries showed a degree of positive correlation with brain derived libraries, heart libraries showed weak positive correlation with muscle libraries and muscle libraries shown some positive correlation with heart libraries (Figure 3). Some positive correlation between these groups of libraries is likely because of the very similar nature of those tissues. But this was unexpected, because one of the original optimisation rounds specifically aimed to exclude such correlation where possible. However such partial positive correlation proves that our EST matrix is also capable of identifying more distant but related tissue types. One particular brain library out of the 13 brain libraries tested (NIH_MGC_181) showed unexpectedly high correlation with pituitary gland. This was much stronger than with the brain expression pattern from the EST expression matrix – the supposed origin of this particular library (Figure 3A). A plausible explanation might be an unintentional inclusion of pituitary gland tissue with the brain tissues for the original library preparation; this is likely due to the close proximity of pituitary gland which is located at the base of the midbrain. Despite the inclusion of this library in the original selection and into the subsequent optimisation steps as “brain” derived, our EST matrix was still able to pick this inaccurately annotated library, thus confirming the robustness of our approach to cluster selection for the EST expression matrix. Using just tissue-specificity (the traditional approach which relies on comparing gene expression between tissues) would have counted such pituitary library as brain derived, which would have influenced the selection of “tissue specific” genes, for which incorrect tissue specificity would have been assigned.

**Figure 4:**
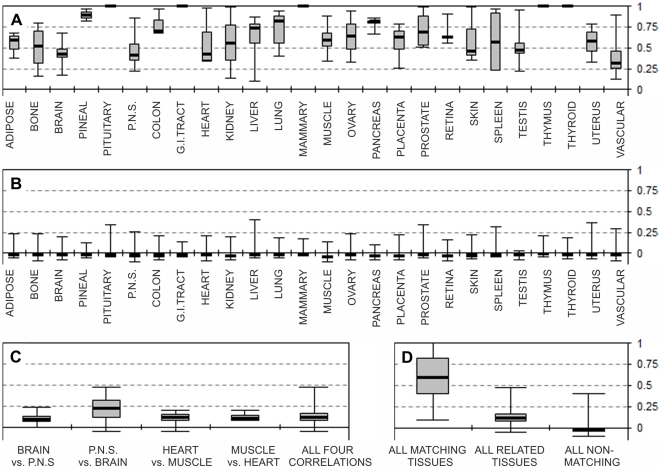
Intra-tissue and inter-tissue correlations. Correlation coefficients calculated for all of the 113 EST libraries (Supplementary Dataset S5) against our EST expression matrix (Supplementary Dataset S4). The data also include the tissues detailed previously in Figures 1–3. **A:** Positive correlations between all expected matching libraries, e.g. all individual “Adipose” libraries vs. the “Adipose” expression matrix (Supplementary Dataset S4) etc. Correlation value of “1” is for tissues where only one EST library was available. **B:** Correlations for all expected non-matching libraries, e.g. all “Adipose” libraries available vs. all but the “Adipose” expression arrays from our EST matrix (Supplementary Dataset S4) etc. The presumed mixed tissue brain library “NIH_MGC_181” was excluded from calculations. **C:** Correlations for all expected related tissues, e.g. all individual “Bain” libraries available vs. the “Peripheral nervous system” expression matrix, etc. **D:** All expected positive correlations from all matching libraries as in panel A (left box plot). Correlations from all related tissues as in panel B (middle box plot). All expected correlations from non-matching tissues, as in panel C (right). In all panels the boxes are drawn from the first to third quartiles. Plots also show minimum value, median (thick line) and the maximum correlation values recorded.

Figure 4A summarises the correlation ranges for all the expected matching tissues, including the tissues detailed in Figures 1, 2, 3. The first and third quartiles for all the positively correlated libraries from all tissues studied are 0.4 and 0.8 respectively (full range 0.2 to 1). The negative inter-tissue correlations are shown in Figure 4B. These values are based on all of the non-matching inter-tissue correlations, with first and third quartile values of −0.04 and −0.02 respectively. The expected inter-tissue correlations, such as brain with peripheral nervous system and heart with muscle) are summarised in Figure 4C. These correlations values are lower than the tissue-specific intra-tissue matches (Figure 4A), but notably higher than correlations between any non-matching tissues (Figure 4B), with the first and third quartiles at ∼0 and +0.14 respectively. Figure 4D compares all three correlations ranges for all cases (positive tissue matches, related tissues, and non-matching tissues).

### EST libraries from mixed, uncharacterised or poorly defined tissue preparations

**Figure 5:**
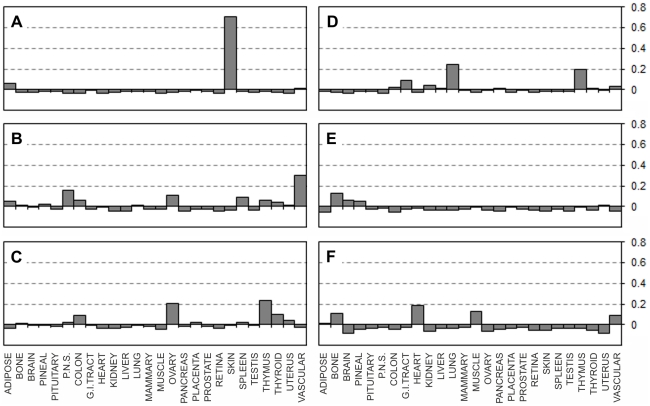
Correlation of the EST matrix with individual libraries from uncharacterised or poorly defined tissue preparations. Pearson correlation coefficients (vertical axes) calculated between the individual EST libraries and the EST expression matrix (Supplementary Dataset S4). **A:** “Uncharacterised” library NCI_CGAP_HN5 derived from gum tissue. **B:** “Uncharacterised” Stratagene endothelial cell 937223 library. **C and D:** pooled libraries NIH_MGC_184 and NCI_CGAP_HN20 respectively. **E:** “Embryo, 8 week I” library. **F:** “Embryo, 12 week II” library.

We further decided to apply our EST expression matrix to the identification of unknown or mixed tissue libraries. A small number of EST libraries annotated as being produced from uncharacterised tissues and therefore not included in our EST selection procedure, but for which their tissues origins are identifiable, were used. Figure 5A shows correlation results for one such library (NCI_CGAP_HN5), derived from gum tissue. This library shows clear positive correlation with the skin tissue type, which is the most related tissue type from the 26 tissue types included in our EST matrix, proving the accuracy of tissue typing using our matrix. Another example of uncharacterised library is the umbilical cord library (Stratagene endothelial cell 937223) which showed positive correlation with vascular tissue type and to a lesser degree with ovary and peripheral nervous system tissue types (Figure 5B). Whilst high positive correlation with vascular tissue and a degree of correlation with the ovary are likely, correlation with peripheral nervous system was unexpected because nervous fibres are only present in the proximal part of the umbilical cord [20]. However, since ovaries are innervated, the matching of both ovary and peripheral nervous system tissue types might be easily explained if the original preparation of umbilical cord contained some ovary tissue. In the absence of further independent information on that library source it would be reasonable to assume that the tissue could have contained some ovary tissue or was obtained form the proximal part of the umbilical cord. However, the highest positive correlation for this EST library is with vascular tissue which is the best match from the tissues available in the matrix. These examples show that our EST expression matrix can help to identify tissue origins of EST libraries. Figure 5C shows an example of correlations obtained for a pooled library (NIH_MGC_184). The correlations indicate the presence of a mixed (lung+thymus) tissues. Such a particular tissue mixture is not impossible, since these two tissues are normally situated in very close proximity to each other and the library may indeed have been made from such a mixed tissue preparation (the library annotation is “pooled tissue”). Another example of mixed tissue library “NCI_CGAP_HN20” is shown in Figure 5D. Correlations indicate the presence of ovary and thymus, the combination which is unlikely to have occurred by accidental tissue mixing, since the two organs are normally located far apart, but the library description does not specify the tissue origins and therefore no means exist to prove or disprove this tissue matching. A conclusion from this particular result would be to avoid using such a library for quantitative expression analysis. Figures 5E and 5F exemplify correlation values obtained for embryonic libraries (“Embryo, 8 week I” and “Embryo, 12 week II” respectively). If these annotations are correct, and both libraries are made from the unfractionated embryonic tissue, our data would suggest that bone and brain tissue markers should have been more prominent at the earlier stages of development whilst towards week 12 muscle specific markers dominate. Such changes do indeed reflect the high prominence of the brain over the rest of the embryo at early gestation stages and the forming of bone around weeks 5 and 10 of gestation [21], followed by the development of muscle tissues and heart at later developmental stages [22], [23] thus validating our interpretation. The stronger correlation with vascular tissue in the 12-week library is consistent with increasing vascularisation following the development of the heart.

### EST libraries from cancer preparations

**Figure 6:**
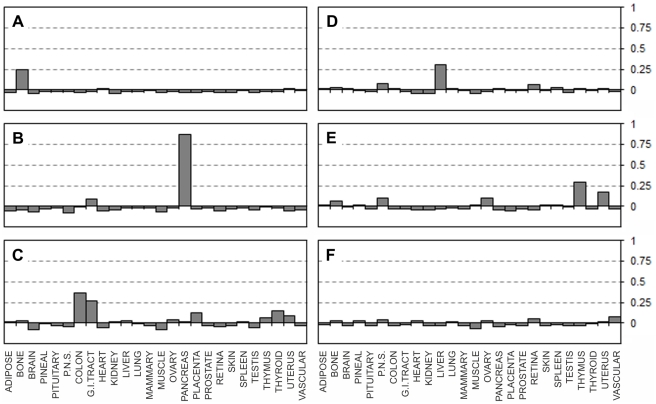
Correlation of the EST expression matrix with individual EST libraries from cancer preparations. Pearson correlation coefficients (vertical axes) calculated between the individual EST libraries and the EST expression matrix (Supplementary Dataset S4). **A:** Bone cancer library NCI_CGAP_Ch1. **B:** Pancreatic library “Pancreas tumor III”. **C:** Colon cancer library NCI_CGAP_Co12. **D:** Brain cancer library NCI_CGAP_Brn64. **E:** Kidney cancer library NCI_CGAP_Kid13. **F:** Brain cancer library NCI_CGAP_Brn53.

Although initial cluster selection procedure relied on both normal and cancer libraries, about 95% of all the UniGene clusters found were present in normal tissues. Our optimisation procedures relied on the normal EST libraries only. It was therefore interesting to see how our EST matrix would score cancer library preparations, which are expected to reflect aberrations in gene expression as well as genomic abnormalities which characterise cancers. Figure 6 shows a few typical examples of correlations obtained for a number of EST libraries from non-normalised bulk cancer tissues; these can be divided in two main categories. The first group represent cancer libraries which correlate well with the stated tissues of origins (Figures 6A, 6B, 6C). One exception is a colon cancer library “NCI_CGAP_Co12”, where “Gastrointestinal tract” EST profile scored nearly as well as the “Colon” profile. We believe this is likely because of the close relation between the two tissue type definitions or because mixed tissue preparation was used, or both. The second group of libraries produced unexpected correlation results (Figures 6D, 6E, 6F). The tissue of origin did not score in any of these, and the matching, at least numerically, was with apparently irrelevant tissues (liver instead of brain in “NCI_CGAP_Brn64”, thymus instead of kidney in “NCI_CGAP_Kid13” and no single tissue scored in brain cancer library “NCI_CGAP_Brn53” (Figure 6F). Clear tissue type matching in some cases of cancer derived libraries, but not in others is probably due to differences in cancer progression. It is reasonable to expect that gene expression changes will increase with the progression of cancer and the progressive deregulation of normal cellular processes. The decreasing accuracy of tissue matching for cancer samples using our EST expression matrix is an indication that the analysis should be capable, in principle, of accurate cancer staging.

### Normalised EST libraries

**Figure 7:**
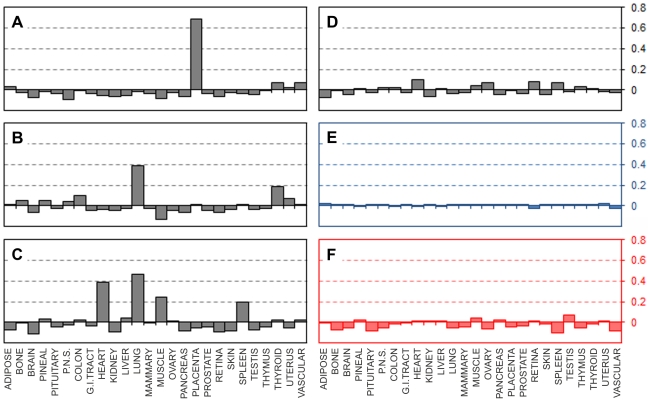
Correlation of the EST expression matrix with normalised EST libraries. Pearson correlation coefficients (vertical axes) calculated between the individual normalised EST libraries, two model libraries and the EST expression matrix (Supplementary Dataset S4). **A:** Normalised placenta library NIH_MGC_148. **B and C:** normalised lung libraries “UI-CF-EC1” and “UI-CF-FN0” respectively. **D:** Normalised thymus library Soares_thymus_NHFTh. **E:** Artificial “normalised” EST matrix where all the expression levels are set to “1” (shown in blue). **F:** Artificial “random” EST matrix where all the expression levels are randomly assigned (shown in red).

Normalising a cDNA library changes the apparent expression levels in that library and should ultimately remove any differences in the gene expression (in normalised libraries) or leave only differentially expressed cDNAs (in subtracted libraries). The progressive disappearance of gene expression differences will depend on the degree of normalisation. It might be reasonable to assume that unless the library is completely normalised the genes which were highly over expressed originally may still have high EST counts, albeit reduced to some degree. For example if a hypothetical library containing three genes with relative abundances 1, 10, 100 is partially normalised to yield e.g. 11, 12 and 13 ESTs or e.g. 1, 2 and 3 ESTs, such three datasets would still correlate well with the original counts (for the above example the correlation would be +0.904 in both cases), and both such “normalised” libraries might both score reasonably well if correlated to EST expression matrix such as created in this work. Although normalisation and subtraction are in essence non-linear transformations we continued using Pearson product-moment correlation coefficient and did not calculate Spearman's and Kendall's correlation coefficients in order to keep the results comparable with all the previous calculations. The correlation data for a number of normalised libraries are shown in Figure 7. Normalised placenta library “NIH_MGC_148” correlated well with placental tissue array from our EST expression matrix scoring (+0.69) despite being normalised (Figure 7A). Two different normalised lung libraries “UI-CF-EC1” and “UI-CF-FN0” both had lung as the most highly positively scored tissue, but had different levels of unanticipated cross-tissues correlation (Figures 7B and 7C). The data in Figure 7C show a degree of positive correlation with heart, muscle and spleen. Such unexpected cross-tissue relations probably arise from gradual loss of lung gene expression specificity following normalisation. This is clearly seen in Figure 7D, where normalised thymus library “Soares_thymus_NHFTh” is scored using our EST matrix. That library correlated with none of the 26 tissue types in our EST matrix.

Using normalised libraries for the selection and optimisation our EST matrix wasn't feasible (with the degree of normalisation unknown no such optimisation was practically achievable). We therefore used alternative approach to validate the lack of tissue correlations found in normalised library such as in Figure 7D. We created an artificial “normalised” EST matrix where all the 244 different UniGene clusters expression levels were set to “1” (except one value set to 0.999 to avoid a divide by zero error in calculating the Pearson correlation coefficient). We then correlated this model “normalised” dataset to out EST expression matrix. Similarly to the normalised thymus library “Soares_thymus_NHFTh”, the artificially “normalised” library did not correlate with any of the other tissues (Figure 7E). Such lack of any correlation between the model “normalised” dataset and any of the tissues confirms that high degree of library normalisation will yield zero correlations if compared with our EST matrix. To further test the robustness of our matrix we created another artificial dataset by assigning random values to each of the 244 UniGene clusters. Such an artificially arbitrary array did not show positive correlation with any of the 26 tissues from our EST expression matrix. A representative graph is shown in Figure 7F. Thus only tissue-specific non-normalised cDNA libraries (such as in Figures 1, 2, 3) are expected and have yielded positive correlations, proving the functionality of our approach.

## Discussion

EST expression data may contain errors originating from many different stages of tissue preparation, mRNA purification, cDNA priming and synthesis, library generation and amplification, DNA sequencing, including the randomness of the clone selection, EST pre-processing, clustering, annotations (ESTs and libraries) as well as data querying, retrieval and processing by databases. Library tissue origins are not always known or correctly reported and some libraries originate from pooled or mixed tissue samples. These can be experimental errors (e.g. inclusion of neighbouring but different and irrelevant tissues or improper separation of dependant and parental tissues) or data entering errors (wrong tissue, wrong field, wrong keywords to name a few). Some of the errors are due to expected variability in conducting experiments, some can be due to human error factors and would be impossible to predict or evaluate. By the time the expression data are made available to the user, many of these issues are difficult, impractical or impossible to check or trace back to the original preparation. Information about cloning and sequencing is provided for some but not all individual cDNA/EST sequences and if available, could be accessed from primary sequence databases (e.g. dbEST division of GenBank) but not from CGAP and other secondary databases. In practice these issues are rarely addressed by tools for digital gene expression analysis, and there is little attempt made by tools available from secondary databases to evaluate the expression data quality. For example, the only statistical analysis currently available from CGAP (http://cgap.nci.nih.gov/cgap.html) calculates a Benjamini Hochberg false discovery rate “Q” value for each UniGene cluster from the difference between the EST counts mapping onto that cluster in two user-selected pools of EST libraries, and even this value seems to be erroneously calculated since it indicates that the probability of the result being a false discovery depends on the display cut-off settings, rather than just the gene expression values themselves. We therefore set out to find ways of evaluating the EST expression data based on the reported expression data alone, by devising a methodology not dissimilar to the use of checksum algorithms for controlling the integrity of data in files, which is independent of the upstream sample processing details of which are often unknown and uncontrollable.

Our results demonstrate that a small EST expression matrix may be used as a tool for confirming tissue specificity of EST libraries of different size, for the quality control of EST expression data or for identifying problems with EST library preparation (mixed tissues, unknown preparation, normalisation) and possibly for providing the additional insight into the disease progression for cancer derived EST libraries.

Recently organism-specific tissue-distribution profiles based on UniGene expression data from GeneBank were reported for a number of different organisms [24]. The main focus of that paper was to overcome natural language variations, aliases and typographical errors when retrieving tissue source information – a common problem of many data repositories, including EST data [17], [25]–[27]. The TissueDistributionDBs allows searching for genes or tissues and calculates a complex tissue specificity indexes for each gene by the use of standardised Tissue Synonym Library and Tissue Ontology data available at BRENDA [28]. The tool is mostly suitable for the analysis of individual genes and can not be used for comparing EST libraries (i.e. collections of genes). No attempt was made to evaluate the suitability of tissue specificity tables for the analysis of EST expression data, no gene subsets were created and genes were not evaluated for their suitability for solving quality control issues and tissue determination. The database appears to have not been updated since 2009.

Analysis of gene expression data for quality control purpose has been attempted previously with SAGE data [29]. Three databases were compared – Gene Expression Atlas (oligonucleotide microarray data), SAGEmap (SAGE libraries) and TissueInfo (EST libraries). Because these databases use different formats for sample annotation and use different statistical methods for data analysis, a method called Preferential Expression Measure (PEM) was devised to score differential expression of genes in libraries grouped into six different tissue categories (brain, kidney, ovary, pancreas, prostate and vascular endothelium) in three databases. Inter-database correlations were measured and were found to be high for brain, prostate and vascular endothelium, but not for kidney, ovary and pancreas. However, inter-library correlations have yet to be applied as a quality control method within one database [29].

In a more recent study, data for 8,570 genes across 46 human tissues from the Gene Expression Omnibus (an Affymetrix microarray data repository) were categorised according to tissue specificity and subcellular localisation of their protein product [30]. The authors reported that widely expressed genes have higher expression levels than genes which are expressed in one or a few tissues. In this respect we are especially pleased to have identified tissue specific genes, which are also highly expressed, contrary to the trend reported in [30].

While many quality control methods were previously suggested, they only focussed on the whole genome [31] or covered aspects of the data such as GC content [1], with few investigations focusing on the tissue-specificity issues [32]. A common shortcoming of many previous reports is that tissue specificity of the genes was reported [33]–[37] but no attempts were made to actually use such data for quality control or evaluation of the expression data. Moreover, even unique “tissue specific genes” might be of little use if they are expressed at low levels and would therefore be absent in many smaller libraries. Furthermore, many existing tools and secondary databases, including the CGAP, are simply sophisticated information retrieval tools, lacking numerical methods for verification of the EST counts and sample origins. The EST counts are assumed to be correct and the libraries to be correctly annotated [38]–[40]. The existing algorithms used to analyse EST expression data place the emphasis on identification of the degree of over/under-expressed for tissue/disease-specific genes by comparing EST counts between two library pools without fully evaluating the quality of the expression data or the origins of the experimental material used, these are simply assumed to be correct and no numerical methods for their verification are made available [38]–[40]. It is not surprising that many such tissue distribution resources are quickly superseded by more recent developments or being taken off-line [27], [41]–[43]. Our approach to the tissue-specificity problem is different from the previously reported attempts in that we looked into origins of the expression data and assessed tissue specificity of the original preparations and the data quality. We were able to generate a small optimised subset of 244 UniGene Cluster IDs which showed high levels of intra-tissue correlation between different EST libraries while presenting low levels of inter-tissue correlation, suggesting high tissue specificity. The reported EST expression matrix can be used to confirm tissue identities of EST expression datasets for all main human tissue types, to provide insight into the origin of uncharacterised libraries, to identify normalised or subtracted libraries or various other experimental artefacts. In a few cases we were able to identify the location of the tumour from which a cancer sample was taken, an extension not previously considered and not previously reported.

The next logical step is to adapt and apply our algorithms to other publicly available gene expression data. We envisage that with the increasing amounts of EST expression data, our optimised EST marker set could be improved and the tissue range might be expanded. The availability of other expression information, such as from SAGE data [44], DNA microarray data [45] and northern blots [46] and merging such data could improve the selection even further. We envisage that the increasing amounts of expression data available could further decrease the number of UniGene IDs in our expression matrix and may allow accurate analysis and tissue typing of the related and dependent tissues.

## Supporting Information

Figure S1 **Correlation between the highly expressed ESTs and the individual EST's maximum counts.** Horizontal axis - the maximum number of times ESTs have been counted in any of the 155 normal non-normalized libraries. Vertical axis - correlation. ESTs which counted at least 18 times in at least one of the libraries are the most resembling of the tissue specific markers identified manually using CGAP tools.
(TIF)

Dataset S1 **The initial selection of UniGene Clusters.** Up to thirty UniGene IDs were selected per each tissue.
(XLS)

Dataset S2 **All the selected UniGene IDs and the non-redundant collection.** All the selected UniGene IDs are combined, sorted (to show differences in the expression of any duplicated IDs in different tissues) and the duplicated UniGene IDs are removed. Tissue identities for the redundant UniGene IDs were assigned based on the highest recorded expression odds ratio.
(XLS)

Dataset S3 **Optimization of UniGene ID selection.** Additional UniGene IDs are added based on the similarity of their maximum recorded expression levels to the maximum recorded levels of the UniGene Clusters form the original election. Optimization of the combined list by two rounds of selective removal of UniGene IDs.
(XLS)

Dataset S4 **EST expression matrix.** Expression levels of the selected 244 marker UniGene IDs in the 26 tissue super libraries.
(XLS)

Dataset S5 **EST libraries tested for inter-tissue correlations.** 113 representative tissue-specific EST libraries tested for inter-tissue correlations using the EST expression matrix.
(XLS)
