# Supplementary figures and images for: The Use of EST Expression Matrixes for the Quality Control of Gene Expression Data
Source: PLoS One. 2012 Mar 8;7(3):e32966. doi: 10.1371/journal.pone.0032966 (PMC3297614; doi:10.1371/journal.pone.0032966)

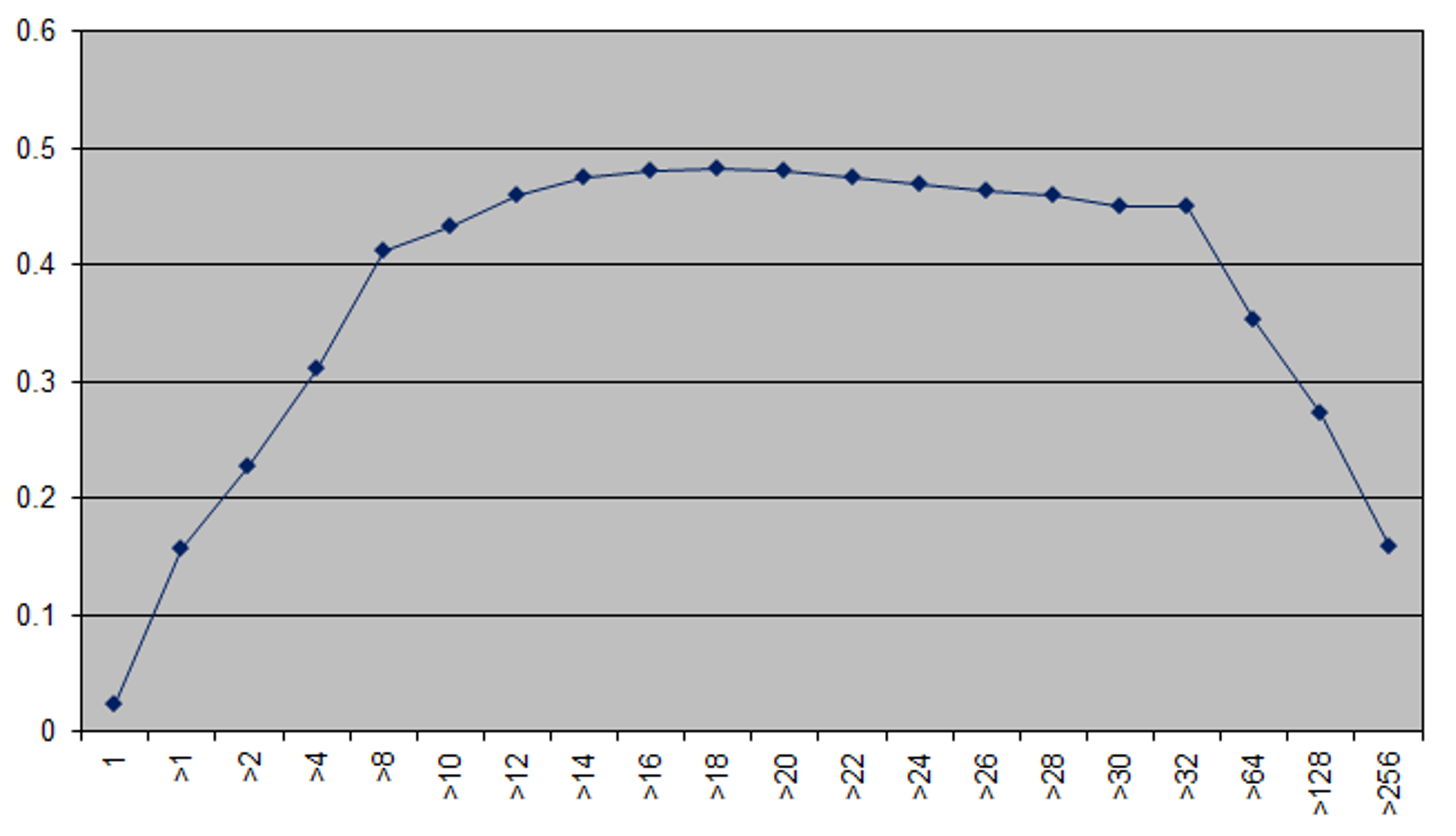

Supplement: Figure S1 — Correlation between the highly expressed ESTs and the individual EST's maximum counts. Horizontal axis - the maximum number of times ESTs have been counted in any of the 155 normal non-normalized libraries. Vertical axis - correlation. ESTs which counted at least 18 times in at least one of the libraries are the most resembling of the tissue specific markers identified manually using CGAP tools. (TIF) [file pone.0032966.s001.tif]
